# Physiological response and molecular regulatory mechanism reveal a positive role of nitric oxide and hydrogen sulfide applications in salt tolerance of *Cyclocarya paliurus*

**DOI:** 10.3389/fpls.2023.1211162

**Published:** 2023-09-01

**Authors:** Lei Zhang, Yang Liu, Zijie Zhang, Shengzuo Fang

**Affiliations:** ^1^ College of Forestry, Nanjing Forestry University, Nanjing, China; ^2^ Co-Innovation Centre for Sustainable Forestry in Southern China, Nanjing Forestry University, Nanjing, China

**Keywords:** *Cyclocarya paliurus*, exogenous substance, photosynthetic parameter, antioxidant system, salt stress, transcription factors

## Abstract

As a multifunctional tree species, *Cyclocarya paliurus* leaves are rich in bioactive substances with precious healthy values. To meet the huge requirement of *C. paliurus* leaf production, sites with some environmental stresses would be potential land for developing its plantations due to the limitation of land resources in China. Nitric oxide (NO) and hydrogen sulfide (H_2_S) are common gas messengers used to alleviate abiotic stress damage, whereas the mechanism of these messengers in regulating salt resistance of *C. paliurus* still remains unclear. We performed a comprehensive study to reveal the physiological response and molecular regulatory mechanism of *C. paliurus* seedlings to the application of exogenous NO and H_2_S under salt stress. The results showed that the application of sodium hydrosulfide (NaHS) and sodium nitroprusside (SNP) not only maintained the photosynthetic capacity and reduced the loss of leaf biomass, but also promoted endogenous NO synthesis and reduced oxidative damage by activating antioxidant enzyme activity and increasing the content of soluble protein and flavonoids. Moreover, transcriptome and metabolome analysis indicated the expression of genes encoding phenylalanine ammonia lyase (PAL), cytochromeP450 (CYP), chalcone synthase (CHS), dihydroflavonol 4-reductase (DFR) and flavonol synthase (FLS) in flavonoid biosynthesis pathway was all up-regulated by the application of NO and H_2_S. Meanwhile, 15 transcriptional factors (TFs) such as WRKY, ERF, bHLH and HY5 induced by NO were found to regulated the activities of several key enzymes in flavonoid biosynthesis pathway under salt stress, via the constructed co-expression network. Our findings revealed the underlying mechanism of NO and H_2_S to alleviate salt stress and regulate flavonoid biosynthesis, which provides a theoretical basis for establishing *C. paliurus* plantations in the salt stress areas.

## 1 Introduction

Salinity is a major environmental factor affecting plant growth and productivity (Guo et al., 2022a). Excessive salt concentration in soil disturbs the osmotic balance, ion transport and metabolic response of plants (Genisel et al., 2015; Mostofa et al., 2015). Salt stress which causes the high concentration of sodium ions in plant cells, not only hinders the absorption of other ions but also interferes with the catalysis of enzyme activity and destroys the nutritional balance (Manishankar et al., 2018). The accumulation of reactive oxygen species such as hydrogen peroxide and superoxide anion in plant cells can seriously damage the structure of various organelles and the biosynthesis of macromolecules (Hazman et al., 2015; Li C. et al., 2017; Ghalati et al., 2020). At present, the area of saline soil in the world has exceeded 800 million hectares and is still expanding (Munns and Tester, 2008). In order to deal with this situation, it is particularly important to reveal the mechanism in response to salt stress and improve the salt tolerance of plants.

Application of exogenous substances on plants is one of the effective methods to alleviate salt stress (Zhang et al., 2019). As important gaseous signal molecules in plants, nitric oxide (NO) and hydrogen sulfide (H_2_S) not only have antioxidant properties, but also widely participate in the whole physiological process from seed germination to plant apoptosis (Singh et al., 2015; Mukherjee, 2019). Moreover, the mechanism of NO and H_2_S in plant cells has also been widely discussed, including their interaction with related active nitrogen substances, modifying target proteins, and regulating intracellular signal transduction (Khurana et al., 2011; Shen et al., 2018). Importantly, many studies have shown that NO and H_2_S are involved in regulating various physiological mechanisms of plants in response to environmental stresses (Begara-Morales et al., 2018; Corpas, 2019; Zhang et al., 2019). For example, NO and H_2_S have been reported to regulate plant response strategies to abiotic stresses such as salinization, drought and cold (Shen et al., 2018; Liu et al., 2020; Zhou et al., 2020). Meanwhile, some studies also indicated that H_2_S increases the content of S-nitrosothiol and then possibly affects the storage abundance of NO (Singh et al., 2015; Mukherjee, 2019) and the crosstalk relationship between NO and H_2_S in cells has become a research hotspot (Singh et al., 2020). For instance, H_2_S has been reported to alleviate salt stress of barley seedling via NO-mediated ion homeostasis (Chen et al., 2015). Another finding showed that NO and H_2_S enhance the resistance of tomato seedlings to heavy metal stress through sulfur assimilation (Alamri et al., 2020). Thus, the application of exogenous NO and H_2_S would be an important way to alleviate abiotic stress of plants, whereas less related information is available in tree species.

*Cyclocarya paliurus*, as a multifunctional tree species in walnut family, is widely distributed in subtropical areas of China (Fang et al., 2006). Especially its leaves are traditionally used for the production of nutraceutical tea and ingredient of functional foods in China because of its unique taste and rich in bioactive substances with hypoglycemic and hypotensive functions (Fang, 2011). Flavonoids is not only an important bioactive substance but also a key antioxidant in plants in response to abiotic stresses (Li X. et al., 2017). Previous studies have showed that salinity increased the flavonoid content of *C. paliurus* leaves (Zhang et al., 2021). Moreover, exogenous H_2_S application has been suggested to induce endogenous NO production, and improve salt tolerance of *C. paliurus* by maintaining fluorescence and enhancing antioxidant activity (Chen et al., 2021). However, the molecular regulation mechanisms of NO and H_2_S applications on *C. paliurus* under salt stress still remain unknown.

To meet the huge requirement of *C. paliurus* leaf production, sites with some environmental stresses would be potential land for developing *C. paliurus* plantations due to the limitation of land resources in China, while the coastal saline land has been regarded as a potential area for developing *C. paliurus* resources. Therefore, how to improve the salt tolerance of *C. paliurus* has become particularly urgent in the practices. Moreover, flavonoid is considered not only to be important bioactive substances but also to resist environmental stresses (Maurya and Yadav, 2005; Guo et al., 2022b), so revealing how exogenous substances affect flavonoid biosynthesis in leaves is also important under the salinity condition. The objectives of this research were: (1) to identify the role of NO and H_2_S on photosynthetic process and antioxidant capacity of *C. paliurus* under salt stress; (2) to explore the regulatory function of NO and H_2_S on *C. paliurus* seedlings in response to salt stress from the perspective of transcriptome and metabolome; (3) to clarify the molecular mechanism of the above signaling molecules acting on the flavonoid pathway. Results from this study may provide new insights into the molecular mechanism of NO and H_2_S on alleviating abiotic stress of woody plants.

## 2 Materials and methods

### 2.1 Plant materials and treatments

*C. paliurus* seeds were collected from Yanling county (26° 30′ N, 113° 41′ E) in Hunan province, China, in October 2018. Based on the method of Fang et al. (2006), seeds were treated with exogenous gibberellin A3 and stratification measures to break seed dormancy. In April of 2019, the germinated seeds were sown in nonwoven receptacles (10.0 cm depth and 8.5 cm diameter) and then transferred to the greenhouse at Baima Experimental Base of Nanjing Forestry University (31° 35′ N, 119° 09′ E). The nonwoven receptacles were filled with mixed substrates of peat: soil: perlite: rotting poultry manure = 4: 2: 2: 2 (v/v/v/v). The contents of total N, total P, total K, and organic matter in the substrates were 72.35, 2.19, 9.55, and 73.3 g/kg, respectively.

Salt treatments were performed in July 2019, and a completely random block design was adopted with three replicates for each treatment, and six plants for each replicate. Referring to previous research (Ma et al., 2019), six treatments were set up, including CK (no salt addition and only irrigation with distilled water), SNP (no salt addition, spraying with 0.25mM sodium nitroprusside (a NO donor), and irrigation with distilled water), NaHS (no salt addition, spraying with 0.5mM sodium hydrosulfide (a H_2_S donor), and irrigation with distilled water), NaCl (0.4% NaCl treatment and irrigation with 0.4% NaCl solution), SNP+NaCl (0.4% NaCl treatment, spraying with 0.25mM SNP and irrigation with 0.4% NaCl solution), and NaHS+NaCl (0.4% NaCl treatment, spraying with 0.5mM NaHS and irrigation with 0.4% NaCl solution). To avoid osmotic shock, NaCl solution was gradually added into soil in five times within two days to reach the expected concentrations, while the concentration was calculated based on soil weight. However, the solutions of SNP and NaHS were evenly sprayed on the adaxial and abaxial surfaces of leaves before one day of the salt treatment until completely wetting the leaves. The nonwoven receptacles were placed in plastic pot and trays to prevent salt loss, and irrigation was performed twice a week to keep the field water capacity at 70% - 75%.

**Figure 1:**
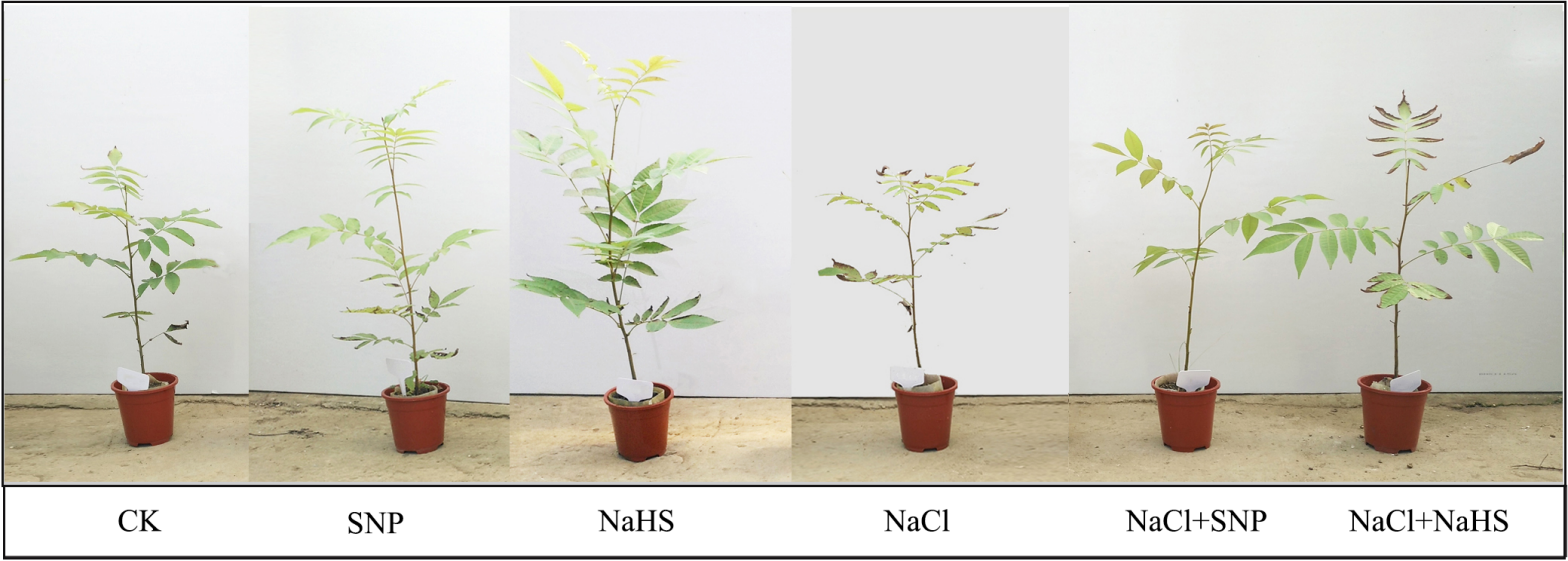
Phenotypes of *C. paliurus* seedlings after 30 days of the treatments. Code annotation: CK (no salt addition and only irrigation with distilled water), SNP (no salt addition, spraying with 0.25mM sodium nitroprusside (a NO donor), and irrigation with distilled water), NaHS (no salt addition, spraying with 0.5mM sodium hydrosulfide (a H_2_S donor), and irrigation with distilled water), NaCl (0.4% NaCl treatment and irrigation with 0.4% NaCl solution), SNP+NaCl (0.4% NaCl treatment, spraying with 0.25mM SNP and irrigation with 0.4% NaCl solution), and NaHS+NaCl (0.4% NaCl treatment, spraying with 0.5mM NaHS and irrigation with 0.4% NaCl solution), the same below.

Leaf samples were collected for the determination of related indexes 30 days after the salt treatments, in that the phenotypic differences were observed among different treatments (**Figure 1**). Three plants of each treatment were selected as samples and six expanded mature leaves were collected from the upper position of each sample. These leaves were quickly frozen by liquid nitrogen and kept at -80°C for total RNA extraction and enzyme activity determination. The rest leaves were dried at 70°C and pulverized for the quantitative analysis of flavonoid content.

### 2.2 Determination of leaf biomass and physiological parameters

#### 2.2.1 Leaf biomass and photosynthetic parameters

Based on the seedling height and ground diameter, three seedlings with uniform growth in each treatment were selected as sample plants for physiological parameters determination. The total leaf biomass was measured by drying the leaves at 70°C for 72 h.

The fourth fully expanded compound leaf of each sample plant was selected to measure photosynthetic parameters by the LI-6400XT photosynthetic system (LI-COR, Inc., Lincoln NE, USA). Photosynthetic parameters including net photosynthetic rate (P_n_), transpiration rate (T_r_), stomatal conductance (G_s_), intercellular CO_2_ concentration (C_i_) and water use efficiency (WUE) were measured between 8:30–11:00 am 30 days after salt treatments. The operating ambient of the photosynthetic apparatus was set according to the method of Zhang et al. (2022).

#### 2.2.2 Fluorescence parameters and chlorophyll content

Fluorescence parameters of the selected leaves were determined by an FMS-2 portable pulse-modulated fluorometer (Hansatech Instruments Ltd., Norfolk, United Kingdom). After 15 minutes of dark adaptation, the selected leaves were used for the determination of maximal quantum yield of PS II (F_v_/F_m_), non-photochemical quenching coefficient (NPQ), and electron transfer rate (ETR).

After determining the photosynthetic and fluorescence parameters, fresh leaves were collected from the same part of the seedlings, washed and dried, and extracted with 80% acetone solution to determine the chlorophyll content following the method of Asghar et al. (2016).

#### 2.2.3 NO and soluble protein content

0.3 g fresh leaf powder was ground with 8 ml double distilled water, and then centrifuged for 20 minutes (4°C, 1,2000×g). The contents of endogenous NO and soluble protein in *C. paliurus* leaves were determined according to the instruction of NO oxide determination kit and soluble protein kit (Jiancheng Bioengineering Institute, Nanjing, China) (Usuda et al., 1984; Cantrel et al., 2010).

### 2.3 Measurement of antioxidant enzyme activity and leaf flavonoid content

#### 2.3.1 Antioxidant enzyme activity

0.3 g fresh leaf samples were homogenized in 50 mM phosphate buffer solution (4.5 ml, containing 0.1 mM ethylene diamine tetra acetic acid and 2 mM dithiothreitol, PH 7.0-7.4). Homogenate was centrifuged at 1,0000×g for 20 min at 4°C, and the supernatant was used for activity analysis of antioxidant enzymes. Antioxidant enzyme activity of *C. paliurus* leaves was detected by a superoxide dismutase (SOD) kit, peroxidase (POD) kit, catalase (CAT) kit, and malondialdehyde (MDA) kit (Jiancheng Bioengineering Institute, Nanjing, China) (Meloni et al., 2003; Rahnama and Ebrahimzadeh, 2005).

#### 2.3.2 Leaf flavonoid content

Alcohol extraction method was adopted to extract leaf samples for the determination of flavonoid content (Cao et al., 2017). In short, 10 mL 70% (v/v) ethanol mixed with 0.8 g dry leaf powder was ultrasonicated 45 min at 70°C for the preparation of extracting solution. The solution was centrifuged at 10,000 rpm for 10 min when cooling to room temperature, and then separated on C18 solid phase extraction column and filtered through a 0.22 µm polytetrafluoroethylene (PTFE) filter. The total flavonoid content was measured by optimized colorimetry (Bao et al., 2005) and the individual flavonoid content was detected by a high-performance liquid chromatography (HPLC) (Waters Crop., Milford, MA, USA) (Cao et al., 2017).

### 2.4 Transcriptomic and metabolomic analysis

#### 2.4.1 Transcriptomic analysis and weighted gene co-expression network construction

Transcriptome sequencing was executed by the method of Guo et al. (2020). In brief, the extraction of total RNA from *C. paliurus* leaves was performed using Trizol reagent kit (Invitrogen, Carlsbad, CA, USA). The cDNA fragments were purified with QiaQuick PCR extraction kit (Qiagen, Venlo, The Netherlands). A total of 18 cDNA libraries (six treatments, and three biological replicates for each treatment) were constructed using Illumina HiSeqTM 4000 by Gene Denovo Biotechnology Co. (Guangzhou, China). High-quality clean reads were acquired with filtering the raw reads by Fastp (Version 0.18.0) (Chen et al., 2018), and the mapped reads of each sample were assembled by StringTie v1.3.1 (Pertea et al., 2015; Pertea et al., 2016). The value of FPKM was calculated by StringTie software (max_memory, 30G; seqType, fq; CPU, 10; KMER_SIZE, 31; min_kmer_cov, 11; normalize_reads; normalize_max_read_cov, 50).

In order to expound the interaction between transcription factors and key structural genes in flavonoid biosynthesis pathway, a weighted gene co-expression network analysis was executed by R packages including WGCNA and DESeq2 software (threshold power = 8, min module size = 50). The Pearson correlations between the eigengenes of each module and the abundance of flavonoids were performed using R package ggplot2. The gene regulatory networks between transcription factors and structural genes were constructed with Cytoscape software (Version 3.7.1) (Shannon et al., 2003).

#### 2.4.2 Metabolomics analysis

The extraction of metabolites for leaf samples was performed by UHPLC-QE-MS (Guo et al., 2020). LC-MS/MS analyses were executed by an UHPLC system (1290, Agilent Technologies) with a UPLC HSS T3 column coupled to Q Exactive (Orbitrap MS, Thermo). The mobile phase proportioning and the elution gradient setting were performed by the method of Zhang et al. (2021). The full scan resolution of ESI source was 70000, and MS/MS used a resolution of 17500. The MS raw data was formatted by ProteoWizard and the conversion results were processed by R package XCMS software (version 3.2). The peak annotation was implemented by OSI-SMMS (version 1.0, Dalian Chem Data Solution Information Technology Co. Ltd.) with in-house MS/MS database. Principal component analysis was performed by R package models for the exposition of relevance among samples. And the metabolites were annotated and classified according to the Kyoto Encyclopedia of Genes and Genomics (KEGG) database.

### 2.5 Statistical analysis

All the statistical analyses were performed using R package models and TBtools software (Version 1.068) (Chen et al., 2020). Differences between samples were determined by one-way analysis of variance (ANOVA) and significant differences were calculated by the least significant difference (LSD) test at *P* < 0.05.

## 3 Results

### 3.1 Effects of exogenous NO and H2S on leaf biomass and physiological parameters under salt stress

**Figure 2:**
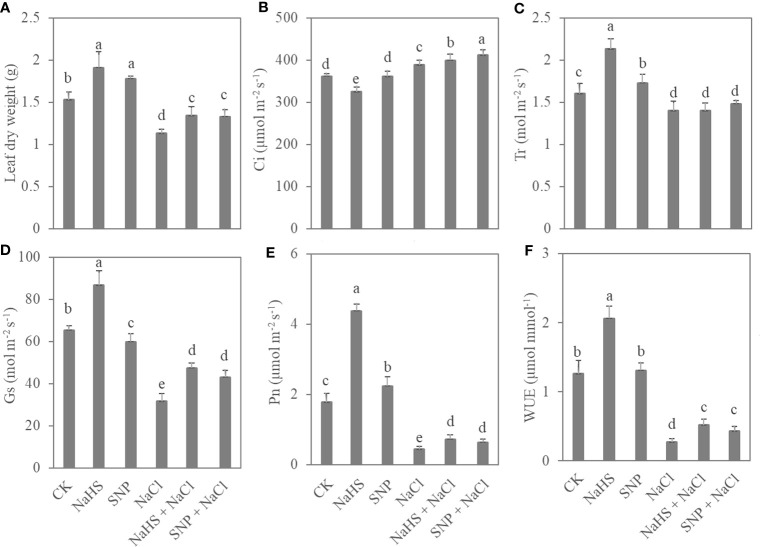
Leaf biomass **(A)** and photosynthetic parameters **(B–F)** in *C. paliurus* seedlings of different treatments. Different small letters indicate a significant difference (*P* < 0.05) among the treatments.

Exogenous NO and H_2_S applications significantly promoted the accumulation of leaf biomass and photosynthetic capacity (**Figure 2**). Compared with the control, NaHS treatment increased leaf dry weight, T_r_, P_n_, and WUE by 25%, 33%, 147%, and 63%, respectively. Moreover, salt treatment caused a significant reduction in leaf biomass and photosynthetic parameters of *C. paliurus* seedlings, whereas the application of exogenous NO and H_2_S alleviated this reduction (*P* < 0.05) (**Figure 2**). In comparison with NaCl treatment, SNP + NaCl treatment increased leaf biomass, P_n_, and WUE of the seedlings by 18%, 47%, and 59%, respectively.

NPQ represents the proportion of light energy dissipated in the form of heat. The value of NPQ significantly enhanced under NaCl treatment in comparison with CK, indicating that the light energy conversion of *C. paliurus* leaves were inhibited by salt, while exogenous NO and H_2_S reduced the NPQ value (*P* < 0.05) (**Table 1**). Compared with salt-treated plants, applications of NaHS and SNP resulted in an increase in ETR (24%-35%) and F_v_/F_m_ (17%-22%) in *C. paliurus* leaves under salinity. Similarly, the contents of chlorophyll a and b increased significantly with the application of exogenous NO and H_2_S (*P* < 0.05) (**Table 1**).

**Table 1:** Variations in fluorescence parameters and chlorophyll content of *C. paliurus* leaves among different treatments.

Treatments	NPQ	ETR	F_v_/F_m_	Chl a content (mg g^-1^)	Chl b content (mg g^-1^)
CK	2.17 ± 0.12 c	0.72 ± 0.09 a	0.70 ± 0.06 ab	0.81 ± 0.05 b	0.38 ± 0.04 b
NaHS	1.96 ± 0.14 c	0.68 ± 0.05 a	0.78 ± 0.02 a	1.12 ± 0.06 a	0.41 ± 0.02 ab
SNP	2.17 ± 0.30 c	0.66 ± 0.08 a	0.76 ± 0.02 a	1.21 ± 0.17 a	0.44 ± 0.02 a
NaCl	3.93 ± 0.44 a	0.37 ± 0.04 c	0.54 ± 0.04 c	0.44 ± 0.01 d	0.20 ± 0.02 c
NaHS+NaCl	3.12 ± 0.47 b	0.46 ± 0.05 bc	0.63 ± 0.09 bc	0.61 ± 0.01 c	0.24 ± 0.03 c
SNP+NaCl	3.31 ± 0.22 b	0.50 ± 0.03 b	0.66 ± 0.05 b	0.66 ± 0.06 bc	0.21 ± 0.01 c

Different small letters indicate a significant difference (P < 0.05) among the treatments. Code annotation: CK (no salt addition and only irrigation with distilled water), SNP (no salt addition, spraying with 0.25mM sodium nitroprusside (a NO donor), and irrigation with distilled water), NaHS (no salt addition, spraying with 0.5mM sodium hydrosulfide (a H_2_S donor), and irrigation with distilled water), NaCl (0.4% NaCl treatment and irrigation with 0.4% NaCl solution), SNP+NaCl (0.4% NaCl treatment, spraying with 0.25mM SNP and irrigation with 0.4% NaCl solution), and NaHS+NaCl (0.4% NaCl treatment, spraying with 0.5mM NaHS and irrigation with 0.4% NaCl solution), the same below.

**Figure 3:**
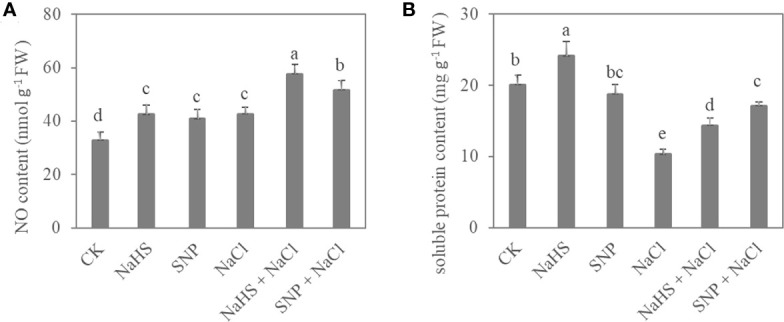
NO content **(A)** and soluble protein content **(B)** in *C. paliurus* leaves of different treatments. Different small letters indicate a significant difference (*P* < 0.05) among treatments.

Exogenous NO and H_2_S also significantly affected the content of NO and soluble protein in *C. paliurus* leaves (*P* < 0.05) (**Figure 3**). Compared with NaCl treatment, the NO content of *C. paliurus* under NaHS+NaCl and SNP+NaCl treatments showed an increase by 35% and 21%, respectively (**Figure 3A**). Moreover, the NaHS and SNP application significantly promoted soluble protein content under salt stress (**Figure 3B**).

### 3.2 NO and H2S applications promote antioxidant enzyme activities and flavonoid contents under salt stress

Salt treatments significantly affected the activities of antioxidant enzymes in *C. paliurus* leaves (**Table 2**). Compared with the control, the activities of POD, SOD, and CAT in the leaves under salt treatment decreased 15%, 48% and 60% respectively, whereas the content of MDA increased significantly under salt stress. However, the activities of antioxidant enzymes in leaves were enhanced by exogenous NO and H_2_S applications (**Table 2**). For example, compared with salt-treated plants, SNP+NaCl treatment significantly promoted the activities of POD (70%), SOD (59%), and CAT (129%) in *C. paliurus* leaves. Besides, the content of MDA under NaHS+NaCl and SNP+NaCl treatments significantly decreased in comparison with NaCl treatment (*P* < 0.05). These results suggested that NO and H_2_S applications can alleviate oxidative stress response under salinity at a certain extent.

**Table 2:** Variations in antioxidant enzyme activity of *C. paliurus* leaves among different treatments.

Treatments	POD activity (U min^-1^ g^-1^)	SOD activity (U g^-1^)	CAT activity (U min^-1^ g^-1^)	MDA content (μmol g^-1^)
CK	25.21 ± 1.02 bc	385.88 ± 9.08 a	6.44 ± 0.27 c	25.03 ± 2.93 c
NaHS	28.89 ± 2.59 b	417.16 ± 5.87 a	8.08 ± 0.43 b	24.39 ± 1.10 c
SNP	34.08 ± 2.55 a	416.39 ± 10.41 a	9.16 ± 0.73 a	27.86 ± 2.55 c
NaCl	21.49 ± 1.22 c	201.20 ± 27.39 c	2.55 ± 0.53 e	74.75 ± 3.11 a
NaHS+NaCl	27.04 ± 1.54 b	349.30 ± 32.83 b	5.08 ± 0.33 d	63.21 ± 3.62 b
SNP+NaCl	36.63 ± 3.69 a	319.13 ± 14.26 b	5.85 ± 0.19 cd	62.33 ± 1.35 b

Different small letters indicate a significant difference (P < 0.05) among the treatments.

As shown in **Table 3**, the contents of total flavonoids and seven individual compounds in the leaves under NaHS and SNP treatments showed an overall upward trend when compared with the control. Although the salt stress treatment significantly increased the content of total flavonoids in comparison with CK (*P* < 0.05), compared with salt stress treatment, the treatments of SNP + NaCl and NaHS + NaCl enhanced the total flavonoid content by 27% and 6% respectively. Similarly, the contents of several individual flavonoid compounds, such as quercetin-3-O-glucuronide, kaempferol-3-O-glucuronide, and kaempferol-3-O-glucoside, were also observed to increase significantly (*P* < 0.05). Thus, our results suggested exogenous NO and H_2_S can enhance flavonoid contents in the leaves under salt stress to alleviate the oxidative damage.

**Table 3:** Variations in flavonoid content among *C. paliurus* leaf samples of different treatments.

Treatments	Flavonoid contents (mg/g)							
	Total flavonoid	Quercetin-3-O-glucuronide	Quercetin-3-O-galactoside	Isoquercitrin	Kaempferol-3-O-glucuronide	Kaempferol-3-O-glucoside	Quercetin-3-O-rhamnoside	Kaempferol-3-O-rhamnoside
CK	39.42 ± 2.21 e	0.21 ± 0.01 d	0.06 ± 0.01 d	0.06 ± 0.01 c	0.45 ± 0.04 d	0.17 ± 0.02 d	0.05 ± 2.81E-03 b	0.13 ± 0.01 c
NaHS	48.82 ± 3.41 d	0.46 ± 0.06 c	0.17 ± 0.02 c	0.15 ± 0.01 b	2.11 ± 0.05 b	0.46 ± 0.02 b	0.06 ± 9.02E-04 b	0.22 ± 0.02 b
SNP	49.32 ± 2.55 d	0.54 ± 0.06 c	0.14 ± 0.03 c	0.14 ± 0.02 b	1.65 ± 0.15 c	0.28 ± 0.03 c	0.05 ± 1.22E-03 b	0.09 ± 0.01 d
NaCl	52.82 ± 4.02 c	0.19 ± 0.01 d	0.06 ± 0.01 d	0.07 ± 0.01 c	0.4 ± 0.04 d	0.16 ± 0.01 d	0.04 ± 2.15E-03 b	0.06 ± 0.01 d
NaHS+NaCl	67.23 ± 3.22 a	0.71 ± 0.05 b	0.44 ± 0.05 a	0.19 ± 0.02 b	2.16 ± 0.29 b	0.31 ± 0.03 c	0.10 ± 9.82E-03 a	0.35 ± 0.01 a
SNP+NaCl	55.94 ± 1.19 b	0.81 ± 0.06 a	0.31 ± 0.04 b	0.54 ± 0.04 a	2.84 ± 0.16 a	0.6 ± 0.04 a	0.08 ± 8.36E-03 a	0.27 ± 0.03 b

Different small letters indicate a significant difference (P < 0.05) among treatments.

### 3.3 Transcriptomic response to NO and H2S applications under salt stress

**Figure 4:**
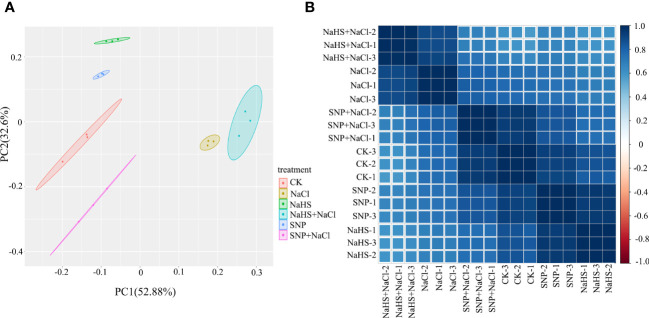
PCA score plot and correlation heat map in transcriptomic profile of *C. paliurus* leaf samples. **(A)** Each point in PCA score plot representing an independent biological replicate; **(B)** Pearson product-moment correlation coefficients of the gene expression profile. The blue rectangles represent the positive correlation between the samples, whereas the red rectangles represent the negative correlation.

The sequencing quality of 18 cDNA libraries of *C. paliurus* leaves were summarized in **Table S1**, and a total of 0.8 billion clean reads were acquired. The proportions of reads mapped to gene of each library ranged from 78.40% to 80.40% (**Table S1**). Through principal component analysis, an obvious separation between samples under different treatments was shown on the plot (**Figure 4A**), indicating that salt treatment and the application of exogenous NO and H2S caused intense recombination at transcriptional level. PC1 and PC2 reflected 52.9% and 32.6% information of gene expression among *C. paliurus* samples, respectively. In addition, Pearson product-moment correlation coefficients showed a close correlation between samples under the same treatment (**Figure 4B**).

**Figure 5:**
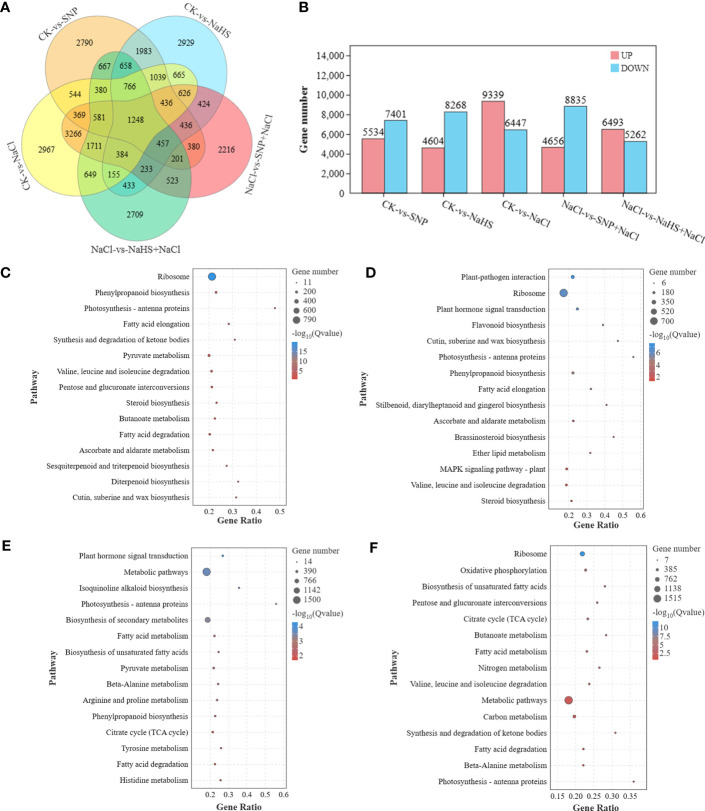
The analysis of DEGs in transcriptomic profile of *C. paliurus* samples and the annotation of KEGG enrichment pathway. Venn diagram **(A)** and the number **(B)** of DEGs between different treatments; Top 15 of KEGG enrichment pathway of DEGs between treatments of CK and SNP **(C)**, CK and NaHS **(D)**, Salt and SNP + NaCl **(E)**, and Salt and NaHS + NaCl **(F)**.

By analyzing the transcriptional profile, a large number of differentially expressed genes (DEGs) were identified from the samples between different treatments (**Figure 5A**). Combined with the results of the five comparison groups, 1248 DEGs were found in all groups, indicating that these genes were susceptible to the factors of salt, NO and H_2_S. However, the largest number of specific DEGs (2967 genes) were identified between CK and salt treatments. After further exploring the differences at transcriptional levels among different treatments, it was found that compared with the CK, a total of 9399 genes were up-regulated and 6447 genes were down-regulated under salt treatment (**Figure 5B**). And the pretreatments of SNP and NaHS before salt treatment resulted in the up-regulation of 4656 and 6493 genes and down-regulation of 8835 and 5262 genes, respectively. According to the enrichment analysis at KEGG pathways, the DEGs between CK and SNP (or NaHS) treatments were found to be enriched in phenylpropanoid biosynthesis, sesquiterpenoid and triterpenoid biosynthesis pathway, and MAPK signaling pathways; and the DEGs between NaCl and SNP + NaCl (or NaHS + NaCl) treatments were enriched at isoquinoline alkaloid biosynthesis, nitrogen metabolism, and flavonoid biosynthesis pathways. In addition, the DEGs among different treatments were collectively enriched in the pathways of ribosome, fatty acid elongation, pyruvate metabolism, citrate cycle and plant hormone signal transduction.

### 3.4 Metabolomic response to NO and H2S applications under salt stress

**Figure 6:**
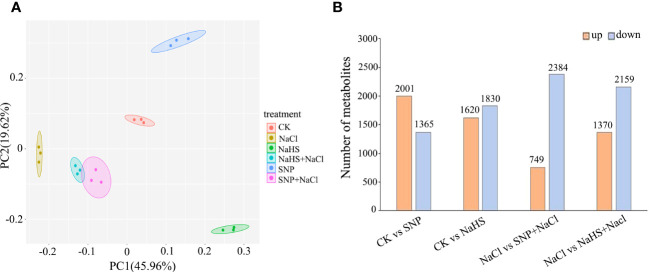
PCA score plot and the number of differentially accumulated metabolites in metabolomic profile of *C. paliurus* leaf samples. **(A)** Each point in PCA score plot representing an independent biological replicate; **(B)** The red and blue columns represent the up-regulation and down-regulation of metabolite abundance, respectively.

As shown in **Figure 6A**, the distribution of samples from different treatments on the PCA diagram of metabolomic data presented a clear separation, which was consistent with the result of the transcriptional analysis. Among the differentially accumulated metabolites between the treatments of NaCl and SNP + NaCl, the number of up-regulation and down-regulation were 749 and 2382, respectively (**Figure 6B**). Similarly, the number of up-regulation was much lower than that of down-regulation of differentially accumulated metabolites between the treatments of NaCl and NaHS + NaCl (**Figure 6B**).

In order to further understand the enrichment information of these metabolites, the top 20 KEGG pathways were shown by bar diagrams (**Figures S1A, B**). Both two diagrams contained the pathways of flavonoid biosynthesis, flavone and flavonol biosynthesis, and anthocyanin biosynthesis.

### 3.5 Metabolite accumulation and gene expression in flavonoid biosynthesis pathway

**Figure 7:**
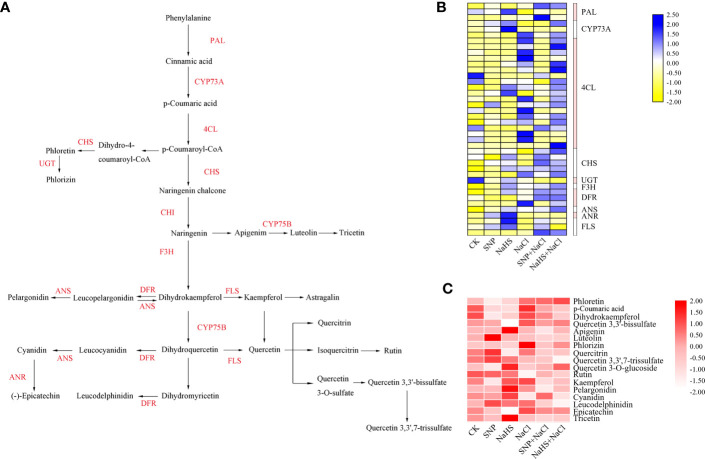
Differential expressions of structural genes and metabolites in flavonoid biosynthesis pathway of *C. paliurus* leaf samples. **(A)** Schematic diagram of metabolic relationship and related genes in flavonoid pathway; **(B)** Blue and yellow blocks represent high and low expression of genes, respectively. Enzyme annotation: 4CL, 4-coumarate: coenzyme A ligase; ANR, Anthocyanidin reductase; ANS, Anthocyanidin synthase; CHI, Chalcone isomerase; CHS, Chalcone synthase; CYP, CytochromeP450; DFR, Dihydroflavonol 4-reductase; F3H, Flavonoid 3-hydroxylase; FLS, Flavonol synthase; PAL, Phenylalanine ammonia lyase; UGT, UDP-glycosyltransferase. **(C)** Heat map representing the abundance of metabolites in the flavonoid pathway. And the shade of red in the blocks represents the abundance of metabolites.

To explore the regulatory effects of NO and H_2_S on flavonoid biosynthesis, a schematic diagram was used to clarify the synthesis relationship from phenylalanine in the upstream to flavonoids in the downstream (**Figure 7A**). A total of 40 DEGs and 17 metabolites with significant differences in abundance between different treatments were screen from the flavonoid biosynthesis pathway (**Tables S2, S3**), and the expression levels of these genes and metabolites were presented by heatmaps (**Figures 7B, C**).

Compared with the CK, the treatment of NaHS significantly up-regulated the expression of related genes encoding PAL (phenylalanine ammonia lyase), CYP (cytochromeP450) and 4CL (4-coumarate: coenzyme A ligase). Moreover, when compared with NaCl, the up-regulated genes under the treatment of NaHS + NaCl ran through the entire pathway, whereas the SNP + NaCl treatment only up-regulated the expression of some downstream genes encoding FLS (flavonol synthase), DFR (dihydroflavonol 4-reductase), and F3H (flavonoid 3-hydroxylase) (**Figure 7B**). Similar to the trend of gene expression, the application of NaHS significantly increased the abundance of a large number of metabolites compared with CK (**Figure 7C**). Notably, the abundance of metabolites under the treatments of SNP + NaCl and NaHS + NaCl decreased when compared with NaCl treatment, suggesting that the large amount of these metabolites may be used for the synthesis of stress resistant substances in the downstream pathways.

### 3.6 Gene correlation networks related to flavonoid biosynthesis

**Figure 8:**
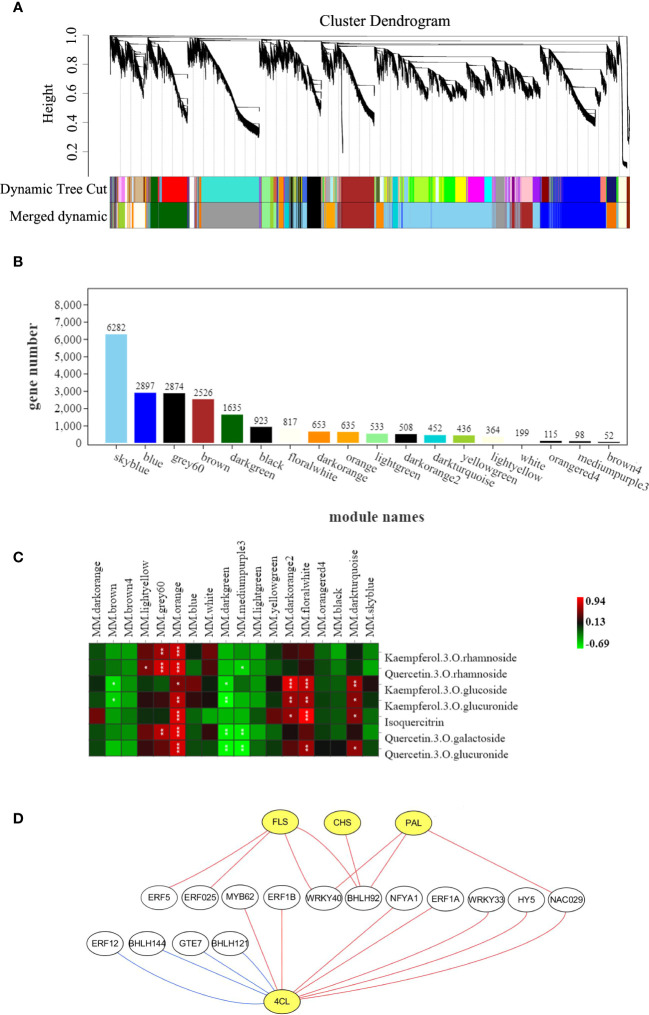
Results of the gene co-expression network analysis. **(A)** Cluster dendrogram showing identified modules and the cluster tree of eigengenes in each module; **(B)** The number of genes in each module; **(C)** The heatmap of correlation coefficient between flavonoid contents and module eigengenes "*", "**" and "***" indicate correlations of 0.5-0.6, 0.6-0.7 and 0.7-1, respectively; with the red and green blocks representing positive and negative correlations, respectively; **(D)** Network constructed by selected TFs (white circles) and structural genes (yellow circles) related to flavonoid biosynthesis pathway from orange module. The red line and blue line represent positive correlation and negative correlation, respectively.

The weighted gene co-expression network analysis (WGCNA) indicated that the genes in transcriptional profile of *C. paliurus* samples were divided into 18 modules based on similar expression patterns (**Figure 8A**). Among them, skyblue module comprised the most genes and brown4 module comprised the least genes, with the number of genes being 6282 and 52 respectively (**Figure 8B**). Pearson correlation analysis showed that orange module was closely correlated with the seven flavonoid compounds (**Figure 8C**), indicating that the eigengenes of this module were related to the accumulation of flavonoids in *C. paliurus* leaves under different treatments. To screen the key regulatory transcription factors (TFs) that may regulate the abundance of flavonoids, a co-expression network based on structural genes and TFs from orange module was constructed (**Figure 8D**). The results showed that four structural genes FLS (Unigene0041673), CHS (Unigene0033841), PAL (Unigene0040065) and 4CL (Unigene0040344) in the flavonoid pathway were regulated by multiple TFs (**Table S4**). In the orange module, the four structural genes were positively correlated with 11 TFs, including *ERF*s, *WRKY*s, *HY5*, and so on. Furthermore, the gene encoding 4CL was negatively correlated with *REF12*, *bHLH144*, *GTE7* and *bHLH121*, indicating that the expression of 4CL was inhibited by these four TFs.

## 4 Discussion

Salt stress, a common abiotic stress in nature, affects agricultural yields significantly, as it can severely inhibit plant growth and development (Ryu and Cho, 2015). Salinity causes osmotic and oxidative stress, interfering with plant photosynthesis (Yang and Guo, 2018). This study indicated that salt stress decreased the net photosynthetic rate, electron transfer rate, chlorophyll content and leaf biomass production of *C. paliurus* (**Table 1** and **Figure 2**). However, the values of P_n_, WUE, ETR, and F_v_/F_m_ of the plants were significantly increased under NaHS+NaCl and SNP+NaCl treatments compared with those under the sole NaCl treatment, suggesting that NO and H_2_S applications play a positive role in regulating photosynthetic rate and light energy conversion efficiency, thus reduce the loss of leaf biomass in salt-treated plants. These results are consistent with previous reports on *Brassica juncea* (Khan et al., 2012), *Glycine max* (Egbichi et al., 2014), and *Matricaria recutita* (Fazelian et al., 2012). Meanwhile, the leaf dry weight, photosynthetic rate and chlorophyll content of the plants under NaHS+NaCl and SNP+NaCl treatments were obviously lower than those in the control (**Table 1** and **Figure 2**), indicating that NO and H_2_S application cannot completely eliminate the negative effects caused by salt stress. Chen et al. (2021) showed that H_2_S application improved biomass accumulation by maintaining photosynthesis, but we did not study the effect of applying concentration on plants under salt stress. Therefore, further research is needed to investigate the regulatory ability of different doses of NO and H_2_S on the salt tolerance of *C. paliurus*

NO and H_2_S are important messengers among plant cells, and can effectively regulate the physiological response under abiotic stress (Jiang et al., 2020; Goyal et al., 2021). The results from this study showed that the application of SNP and NaHS significantly increased the contents of NO and soluble protein in leaves of *C. paliurus* (**Figure 3**), inferring a positive regulatory relationship between nitric oxide and protein content. Similarly, previous studies (Chen et al., 2015; Ma et al., 2019) have shown that H_2_S-induced NO has a positive regulatory effect on plant protein production and ion homeostasis. Antioxidant enzyme system is activated under salt stress to reduce the accumulation of reactive oxygen species (ROS), while the activities of antioxidant enzymes reflect the ability of plants to cope with the stress (Jin et al., 2013). Our results also showed that the activities of POD, SOD, and CAT under 0.4% NaCl treatment were significantly lower than those in the control group (**Table 2**), indicating that excessive salinity stress may destroy the normal physiological process and then decrease enzyme activity (Zhang et al., 2021). However, our results showed that the activities of POD, SOD, and CAT were significantly improved with the application of NO and H_2_S donors (**Table 2**). The results from this study also confirm the hypotheses that exogenous NO and H_2_S can induce the increase of endogenous NO content and ameliorate salt tolerance of plants (Chen et al., 2021). In addition, NO and H_2_S were assumed to mediate antioxidant enzyme activity through persulfidation or S-nitrosation, respectively (Singh et al., 2017; Alamri et al., 2020), however, the translational modifications in these potential ways are need to be further investigated in *C. paliurus*.

Flavonoids are a kind of antioxidants and stress resistant substances in plants (Fini et al., 2011). A large number of studies have shown that plants can promote the production of phenolic metabolites to alleviate salt damage (Hejazi-Mehrizi et al., 2012; Oliveira et al., 2020), which is consistent with our results (**Table 2**). Here, our result suggested that the contents of total flavonoid and several individual compounds such as kaempferol-3-O-glucoside, quercetin-3-O-rhamnoside and kaempferol-3-O-rhamnoside were the highest under the treatments of SNP + NaCl and NaHS + NaCl (**Table 2**). These findings indicate that exogenous NO and H_2_S may regulate the quantity and composition of flavonoid substances to improve the tolerance to salt stress. Some recent reports have indicated that application of SNP and NaHS to stressed plants resulted in an increase of flavonoid content (Li X. et al., 2017; Chen et al., 2021). Therefore, exogenous NO and H_2_S applications may not only be a good way to alleviate abiotic stress, but also contribute to the accumulation of flavonoids in *C. paliurus* leaves.

As a small gas signaling molecular, NO plays a key role in alleviating abiotic stress such as drought, low temperature and salt (Zhao et al., 2004; Abat and Deswal, 2009; Yu et al., 2014). Due to the important functions of NO in plant physiological processes, researches on its regulation mechanism at the molecular level have attracted extensive attention (Imran et al., 2018). In the present study, both salt stress and exogenous NO and H_2_S application all led to obviously transcriptional reprogramming in *C. paliurus* leaves (**Figures 4A, B**), whereas a lots of identified DEGs were enriched in the metabolite pathways such as phenylpropanoid biosynthesis, diterpenoid biosynthesis and flavonoid biosynthesis (**Figures 5C–F**). It has also been reported that NO mediates the up-regulation of genes related to the metabolism of isoflavonoid, terpenoid and cyanoamino acid in *Medicago sativa* under abiotic stress (Zhao et al., 2019). Furthermore, our results showed the application of SNP and NaHS promoted the activation of plant hormone signal transduction pathway and MAPK signaling pathway (**Figures 5C–F**). This indicated that NO and H_2_S could effectively regulate the expression of genes related to pathways of hormone signaling and flavonoid biosynthesis, thereby improving the content of related hormones and flavonoids. In consistent with previous reports (Yu et al., 2014), exogenous NO and H_2_S applications enhance plant tolerance to abiotic stress by mediating a series of physiological and metabolic processes.

As reported, the biosynthesis of various flavonoid metabolites is regulated by PAL, CYP, 4CL and other key enzymes, while the activities of these enzymes are activated or inhibited by a variety of TFs when subjected to diverse stress or environmental factors (Ren et al., 2019; Xu et al., 2020; Qi et al., 2022). Our study found that exogenous NO and H_2_S applications enhanced the activities of several enzymes in the flavonoid pathway (**Figure 7B**), and four genes encoding PAL, 4CL, CHS and FLS were identified to be close correlated with 15 TFs (**Figure 8D**). Interestingly, most of these TFs, including *ERF*, *MYB*, *WRKY*, *bHLH*, and *HY5*, have been reported to be induced by NO and to be involved in stress responses to various adversity (Imran et al., 2018; Wen et al., 2021).

Overexpression of *WRKY* has been reported to enhance salt tolerance, up-regulate the expression of FLS, DFR, and F3H, and increase accumulation of flavonoid and anthocyanins (Wang et al., 2017; Wang N. et al., 2018). In this research, WRKY TF, one of the factors contributing to increasing flavonoid content, was also observed to promote the up-regulation of genes encoding PAL, 4CL and FLS (**Figure 8D**). Meanwhile, HY5 is often described to react to light signal and regulate the biosynthesis of flavonoids such as anthocyanins (An et al., 2019), and has recently been suggested to respond to abiotic stresses such as cold and salt (Wang F. et al., 2018; Kovács et al., 2019). In addition, *ERF*, *bHLH*, and *NAC* have also been reported to regulate the expression of structural genes in the flavonoid pathway (Guo et al., 2020; Premathilake et al., 2020) and our results support the viewpoints mentioned above. Notably, *GTE* was observed to inhibit the expression of 4CL in the present study, whereas less information on its related researches is available and more studies on its internal regulatory mechanism on abiotic stress are required. Moreover, it should be aware that both

NO and H_2_S are dose-dependent signal molecules, and the single concentration applied in this study may lead to a deviation in the summary of regulatory roles. Therefore, further detailed studies are needed to reveal the signaling pathways and molecular mechanisms of these two gas molecules in mediating plant growth and development under salt stress. However, the effects of NO and H_2_S on soil physicochemical properties and soil microbial communities were not explored in this experiment. SNP treatment has been reported to reduce phytophagous nematode density and soil pH (Gao et al., 2011), while the effects of these factors on the salt tolerance of *C. paliurus* need to be further studied.

## 5 Conclusion

To sum up, applications of exogenous NO and H_2_S under salt stress can promote endogenous NO synthesis, reduce oxidative damage via activating antioxidant enzyme activities and increasing the contents of soluble protein and flavonoids, and thus maintain the photosynthetic capacity of *C. paliurus* seedlings. With the application of the gas messengers (NO and H_2_S) under salt stress, the physiological activities and metabolic reactions of the seedlings underwent obvious reprogramming at the molecular level. The messengers activated or inhibited the activity of key enzymes in flavonoid pathway by inducing the expression of TFs such as WRKY, and then affect the accumulation of flavonoid compounds. From the views of physiological response and integrative analysis of metabolome and transcriptome, our study suggests that NO and H_2_S applications show a positive role in salt tolerance of *C. paliurus*, even if more researches are required to reveal insight into its molecular mechanism and be verified in the field trial.

## Data availability statement

The original contributions presented in the study are publicly available. This data can be found here: National Center for Biotechnology information (NCBI), https://www.ncbi.nlm.nih.gov/bioproject/, PRJNA799813.

## Author contributions

LZ: Formal analysis, Data curation, Methodology, Writing - Original Draft, Visualization; YL: Supervision; ZZ: Investigation; SF: Conceptualization, Writing - Review & Editing, Funding acquisition, Project administration.
