# Supplementary material for: Physiological response and molecular regulatory mechanism reveal a positive role of nitric oxide and hydrogen sulfide applications in salt tolerance of Cyclocarya paliurus
Source: Front Plant Sci. 2023 Sep 1;14:1211162. doi: 10.3389/fpls.2023.1211162 (PMC10502730; doi:10.3389/fpls.2023.1211162)
Supplement: Supplementary file 1 [file DataSheet_1.docx]

Supplementary Material

Physiological response and integrative analysis of metabolome and transcriptome reveal a positive role of nitric oxide and hydrogen sulfide applications in salt tolerance of *Cyclocarya paliurus*

Lei Zhang, Yang Liu, Zijie Zhang, Shengzuo Fang ^*^

*** Correspondence:** Dr. Shengzuo Fang: [fangsz@njfu.edu.cn](mailto:fangsz@njfu.edu.cn)

# Supplementary Tables and Figures

**Table S1.** Summary of the sequencing quality of 18 RNA libraries of *C. paliurus* leaves.

| Treatment | Raw reads | Clean reads | Total mapped reads | Reads mapped to gene (%) | Q20 (%) | Q30 (%) | GC (%) | RIN value | N50 number | N50 length |
| --- | --- | --- | --- | --- | --- | --- | --- | --- | --- | --- |
|  |  |  |  |  |  |  |  |  |  |  |
| CK | 48,371,243 | 48,318,088 | 38,338,942 | 79.34 | 98.18 | 94.59 | 47.42 | 6.8 | 21,229 | 1,228 |
| NaCl | 46,240,380 | 46,181,029 | 36,359,871 | 78.73 | 97.68 | 93.46 | 47.79 | 7.0 |  |  |
| SNP | 43,829,101 | 43,762,213 | 34,924,707 | 79.81 | 97.59 | 93.30 | 47.56 | 7.5 |  |  |
| SNP+NaCl | 47,679,261 | 47,614,608 | 38,191,005 | 80.21 | 97.63 | 93.28 | 47.63 | 7.1 |  |  |
| NaHS | 40,010,007 | 39,955,873 | 32,129,915 | 80.40 | 97.80 | 93.74 | 47.43 | 6.4 |  |  |
| NaHS+NaCl | 41,778,914 | 41,728,769 | 32,726,282 | 78.40 | 97.85 | 93.83 | 48.07 | 7.2 |  |  |

**Table S2.** The differentially expressed genes in flavonoid biosynthesis pathway.

| Gene ID | Symbol | **Log2_FC** | | | |
| --- | --- | --- | --- | --- | --- |
|  |  | CK vs SNP | CK vs NaHS | NaCl vs SNP+NaCl | NaCl vs NaHS+NaCl |
|  |  |  |  |  |  |
| Unigene0040065 | PAL | -0.75 | 0.12 | 1.13* | 0.86 |
| Unigene0024749 | PAL | 0.27 | 0.79 | 1.11* | 0.82 |
| Unigene0059047 | PAL | -0.30 | 1.55 | 2.04* | -0.03 |
| Unigene0033849 | CYP | -1.14* | 1.98* | 1.32 | 4.93* |
| Unigene0103989 | CYP | -3.37* | 7.98* | 0.00 | 20.73 |
| Unigene0058935 | CYP | -0.60 | -0.15 | -1.71* | -0.64 |
| Unigene0021572 | 4CL | 0.32 | -20.15 | -5.04* | -0.81 |
| Unigene0026243 | 4CL | -18.35 | 0.00 | -22.17* | 1.90* |
| Unigene0093567 | 4CL | 19.98 | -19.98 | -22.23* | -1.08 |
| Unigene0119564 | 4CL | 0.00 | 0.00 | -22.22* | -3.11 |
| Unigene0036303 | 4CL | 0.00 | 20.64 | -21.77* | 0.78 |
| Unigene0027963 | 4CL | -18.61 | 0.00 | -2.83* | 1.81 |
| Unigene0045399 | 4CL | 22.01* | -22.01* | 20.67 | 0.00 |
| Unigene0036302 | 4CL | 1.08* | -0.53 | -1.04 | 0.51 |
| Unigene0093403 | 4CL | -0.45 | 1.79* | -0.32 | 0.42 |
| Unigene0062012 | 4CL | -0.73 | 1.89* | 2.25* | 2.81* |
| Unigene0085004 | 4CL | 2.17 | -20.78* | -4.78* | -0.89 |
| Unigene0040345 | 4CL | -1.19* | 0.35 | 0.01 | 0.34 |
| Unigene0081554 | 4CL | 21.71* | -21.71* | -23.44 | -3.37* |
| Unigene0079921 | 4CL | -1.27* | 0.25 | -1.80* | -0.50 |
| Unigene0079922 | 4CL | -1.53* | 1.96* | -1.17* | 0.41 |
| Unigene0090350 | 4CL | 2.04 | -2.49* | 1.86* | 1.35 |
| Unigene0081553 | 4CL | 21.27 | -21.27 | -23.77* | -4.29* |
| Unigene0070681 | 4CL | 20.58 | -20.58 | -21.75* | -21.75* |
| Unigene0073824 | 4CL | 0.00 | 0.00 | 0.00 | 22.26* |
| Unigene0079905 | CHS | 0.26 | 0.06 | 2.23* | 2.91* |
| Unigene0122689 | CHS | 1.34* | 0.37 | 1.02 | 0.57 |
| Unigene0033841 | CHS | -1.89* | 2.02* | 2.60* | 1.88* |
| Unigene0088196 | CHS | -1.15* | 2.41* | 0.07 | 0.32 |
| Unigene0068658 | CHS | -0.13 | -0.50 | -1.29* | -0.09 |
| Unigene0039389 | UGT | 1.55* | -0.80 | -0.93 | -1.15 |
| Unigene0020155 | F3H | -0.63 | 1.42* | 0.11 | 0.46 |
| Unigene0093784 | DFR | -0.56 | 1.23* | 0.53 | 0.48 |
| Unigene0109023 | DFR | 1.81 | -2.66* | 1.03 | 1.02 |
| Unigene0012675 | DFR | 0.00 | 0.00 | -23.11* | -1.37 |
| Unigene0027516 | ANS | -1.21* | 1.91* | 0.12 | 0.90 |
| Unigene0086564 | ANR | -0.81 | 1.52* | 0.13 | 0.07 |
| Unigene0041674 | FLS | -1.81* | 4.91* | 6.00* | 4.61* |
| Unigene0037836 | FLS | -1.54* | 2.97* | 3.78* | -23.25* |
| Unigene0041673 | FLS | -0.32 | 0.14 | 2.70* | 2.11* |

* indicates significant differences (*P* < 0.05).

**Table S3.** Difference analysis of metabolite abundance.

| Metabolites | **Log2_FC** | | | |
| --- | --- | --- | --- | --- |
|  | CK vs SNP | CK vs NaHS | NaCl vs SNP+NaCl | NaCl vs NaHS+NaCl |
|  |  |  |  |  |
| Apigenin | -0.05 | 1.65* | -0.81 | 0.02 |
| Cyanidin | -0.10 | 0.45 | 1.10* | 0.09 |
| Dihydrokaempferol | -2.04* | -1.56* | -0.57 | -1.47* |
| Epicatechin | -1.27* | -3.11* | -0.97 | -1.02* |
| Kaempferol | -0.86 | 0.24 | -1.02* | -0.71 |
| Leucodelphinidin | 1.66* | 1.07* | -1.93* | -3.07* |
| Luteolin | 1.44* | -0.05 | -0.3 | -0.25 |
| p-Coumaric acid | -1.94 | -2.07 | -1.41* | -2.09* |
| Pelargonidin | -0.03 | 2.33* | -2.03* | -0.74 |
| Phloretin | -1.45* | -0.79 | 0.11 | 0.52 |
| Phlorizin | 0.09 | -0.22 | -2.23* | -1.45* |
| Quercetin 3,3',7-trissulfate | 1.07* | -0.02 | -0.48 | -2.54* |
| Quercetin 3,3'-bissulfate | -0.12 | -1.52* | -0.5 | -0.31 |
| Quercetin 3-O-glucoside | -1.03 | 2.18* | 0.53 | 1.65 |
| Quercitrin | 0.43* | -1.03* | -0.68 | -0.58 |
| Rutin | -0.19 | -0.54* | 2.37* | 1.35 |
| Tricetin | -0.54 | 1.42* | -0.46 | -0.25 |

*indicates significant differences (*P* < 0.05).

**Table S4.** The identified transcription factors in the flavonoid-related module.

| Gene ID | Name | Annoation |
| --- | --- | --- |
| Unigene0084556 | NAC029 | NAC transcription factor 29-like [*Juglans regia*] |
| Unigene0050194 | WRKY40 | probable WRKY transcription factor 40 [*Juglans regia*] |
| Unigene0055403 | BHLH92 | transcription factor bHLH92-like [*Juglans regia*] |
| Unigene0044852 | ERF1A | ethylene-responsive transcription factor 2-like [*Juglans regia*] |
| Unigene0060998 | ERF12 | ethylene-responsive transcription factor 12-like [*Juglans regia*] |
| Unigene0042239 | NFYA1 | nuclear transcription factor Y subunit A-1-like isoform X1 [*Juglans regia*] |
| Unigene0118059 | MYB62 | transcription factor MYB108-like [*Juglans regia*] |
| Unigene0072225 | HY5 | transcription factor HY5 [*Ziziphus jujuba*] |
| Unigene0046995 | ERF1B | ethylene-responsive transcription factor 1B-like [*Juglans regia*] |
| Unigene0122273 | BHLH144 | transcription factor bHLH144-like [*Juglans regia*] |
| Unigene0002079 | WRKY33 | probable WRKY transcription factor 33 [*Juglans regia*] |
| Unigene0018208 | GTE7 | transcription factor GTE2-like [*Juglans regia*] |
| Unigene0027526 | BHLH121 | transcription factor bHLH121-like isoform X1 [*Juglans regia*] |
| Unigene0078380 | ERF5 | ethylene-responsive transcription factor ERF105-like [*Juglans regia*] |
| Unigene0114889 | ERF025 | ethylene-responsive transcription factor ERF027-like [*Juglans regia*] |


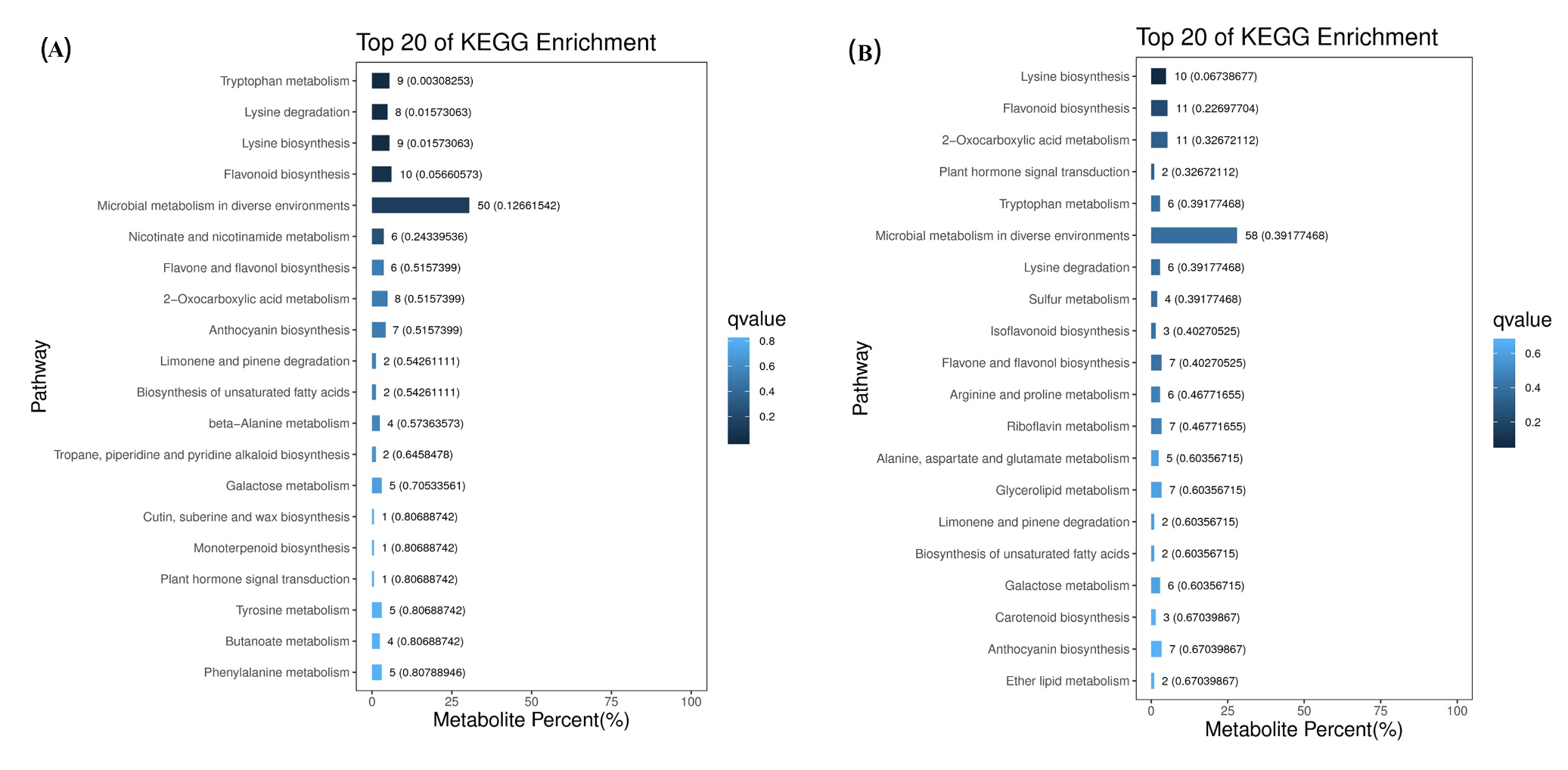


**Figure S1.** KEGG enrichment bar diagrams in metabolomic data of *C. paliurus* leaf samples. (A) KEGG enrichment annotation of differentially accumulated metabolites between the treatments of NaCl and SNP + NaCl; (B) KEGG enrichment annotation of differentially accumulated metabolites between the treatments of NaCl and NaHS + NaCl.
